# Pediatric defibrillation after cardiac arrest: initial response and outcome

**DOI:** 10.1186/cc5005

**Published:** 2006-08-01

**Authors:** Antonio Rodríguez-Núñez, Jesús López-Herce, Cristina García, Pedro Domínguez, Angel Carrillo, Jose María Bellón

**Affiliations:** 1 Pediatric Emergency and Critical Care Division, Department of Pediatrics, Hospital Clínico Universitario de Santiago de Compostela, Servicio Galego de Saúde (SERGAS) and University of Santiago de Compostela, Santiago de Compostela, Spain; 2 Pediatric Intensive Care Unit, Hospital General Universitario Gregorio Marañón, Madrid, Spain; 3 Pediatric Intensive Care Unit, Hospital Infantil Vall d'Hebrón, Barcelona, Spain; 4 Preventive Medicine Service, Hospital General Universitario Gregorio Marañón, Madrid, Spain

## Abstract

**Introduction:**

Shockable rhythms are rare in pediatric cardiac arrest and the results of defibrillation are uncertain. The objective of this study was to analyze the results of cardiopulmonary resuscitation that included defibrillation in children.

**Methods:**

Forty-four out of 241 children (18.2%) who were resuscitated from inhospital or out-of-hospital cardiac arrest had been treated with manual defibrillation. Data were recorded according to the Utstein style. Outcome variables were a sustained return of spontaneous circulation (ROSC) and one-year survival. Characteristics of patients and of resuscitation were evaluated.

**Results:**

Cardiac disease was the major cause of arrest in this group. Ventricular fibrillation (VF) or pulseless ventricular tachycardia (PVT) was the first documented electrocardiogram rhythm in 19 patients (43.2%). A shockable rhythm developed during resuscitation in 25 patients (56.8%). The first shock (dose, 2 J/kg) terminated VF or PVT in eight patients (18.1%). Seventeen children (38.6%) needed more than three shocks to solve VF or PVT. ROSC was achieved in 28 cases (63.6%) and it was sustained in 19 patients (43.2%). Only three patients (6.8%), however, survived at 1-year follow-up. Children with VF or PVT as the first documented rhythm had better ROSC, better initial survival and better final survival than children with subsequent VF or PVT. Children who survived were older than the finally dead patients. No significant differences in response rate were observed when first and second shocks were compared. The survival rate was higher in patients treated with a second shock dose of 2 J/kg than in those who received higher doses. Outcome was not related to the cause or the location of arrest. The survival rate was inversely related to the duration of cardiopulmonary resuscitation.

**Conclusion:**

Defibrillation is necessary in 18% of children who suffer cardiac arrest. Termination of VF or PVT after the first defibrillation dose is achieved in a low percentage of cases. Despite a sustained ROSC being obtained in more than one-third of cases, the final survival remains low. The outcome is very poor when a shockable rhythm develops during resuscitation efforts. New studies are needed to ascertain whether the new international guidelines will contribute to improve the outcome of pediatric cardiac arrest.

## Introduction

Cardiac arrest (CA) in children is typically due to asystole or pulseless electrical activity, whereas ventricular fibrillation (VF) and pulseless ventricular tachycardia (PVT) – namely, shockable rhythms – are relatively rare [1-3]. It has been reported in approximately 8–18% of children with cardiorespiratory arrest that the first documented rhythm is a shockable one [3-11]. A recent multicenter registry identified VF or PVT in 27% of patients with inhospital (IH) CA [12].

Despite extensive experience in adults indicating the first documented electrocardiogram rhythm as a major prognostic factor, there have been very few studies assessing the results of defibrillation in children [12-14]. Reports indicate that adult patients with VF or PVT treated with electric shocks have better outcome that those with asystole or pulseless electrical activity [1-3]. Some pediatric studies, however, did not confirm these results [4,5,9,12].

The optimal defibrillation dose in children is unknown; recommended energy doses for children are derived from limited animal studies [15], from case series with few patients [16], and from extrapolation of adult doses. Studies that prospectively evaluate the effectiveness of current recommendations for pediatric shock doses are lacking, and the data obtained from pediatric animal models [17] and from a case series [13] indicate that a 2 J/kg dose is at least suboptimal. It has been suggested that high shock doses are effective and well tolerated by pediatric hearts [18]. In this sense, the European Resuscitation Council's new guidelines recommend 4 J/kg as the first energy dose for defibrillation in children [19].

The objective of the present study was to evaluate the initial response to defibrillation attempts and the outcome in children with CA, in a prospective, multicenter, Utstein style report of pediatric cardiopulmonary arrest.

## Patients and methods

This is a secondary analysis of data from a prospective study of IH and out-of-hospital (OOH) pediatric cardiopulmonary arrest in Spain that recruited patients from 1 April 1998 to 30 September 1999, the methodology and primary results of which have been described elsewhere [5,20]. A protocol was drawn up in accordance with the Utstein style guidelines. Institutional Review Board approval and parental consent were obtained in each center. Patients aged from seven days to 18 years were eligible for the study if they had presented with CA and defibrillation had been attempted. CA was defined as the inability to palpate a central pulse, unresponsiveness and apnoea, or severe bradycardia lower than 60 beats/minute with poor perfusion in infants requiring external cardiac compressions and assisted ventilation [5,21]. Neonates admitted to neonatal intensive care units were excluded.

The analyzed data included patient-related variables (age, sex, weight, cause of arrest, and personal background), arrest-related and life-support-related variables (type of arrest, location of arrest, monitored parameters, assisted ventilation and/or vasoactive drugs administered before the arrest, time elapsed from the arrest to the start of cardiopulmonary resuscitation (CPR), persons who performed the CPR maneuvers and procedures, the first documented electrocardiogram rhythm, the number and doses of electric shock, and the total duration of CPR), and outcome-related variables (ROSC, initial survival (defined as ROSC maintained for more than 20 minutes), and final survival (defined as survival at one year)). The treatment protocol consisted of the recommendations for CPR released by the Spanish Paediatric Resuscitation Working Group following the international guidelines available at the time of the study [5]; the recommended defibrillation energy doses for the first three shocks at that time were 2 J/kg, 2 J/kg, and 4 J/kg. All shocks were delivered by the manual defibrillators with monophasic waveforms that were available at the time.

### Statistical analysis

Statistical analysis was performed by means of version 12 of the SPSS software statistical program (SPSS Inc. Chicago, Illinois, USA). Pearson's chi-squared test was used for qualitative variables analysis, and Fisher's exact test was used when *n *(number of data) was less than 20 or when any value was less than 5. Student's *t *test was used to compare quantitative variables between independent groups, and the Mann–Whitney *U *test was used for variables not normally distributed. Results are presented as the mean ± standard deviation the median, or the number (percentage). *P *< 0.05 was considered significant.

## Results

Forty-four (28 boys and 16 girls) out of 241 children (18.2%) who suffered IH CA (22 cases) or OOH CA (22 cases) received at least one electric shock. The mean age of the patients was 78.2 ± 66.7 months (range, 1 month–16 years) and the mean weight was 24.8 ± 19.0 kg (range, 3–70 kg).

Patients' characteristics are summarized in Table 1. CA was identified by health professionals in 38 patients (86.4%) and by paramedics in six cases (13.6%). Twenty-five patients (56.8%) were monitored when they suffered the CA episode, 20 patients (45.5%) were on mechanical ventilation, and 16 patients (36.4%) were treated with vasoactive drugs at the time of CA. The time elapsed from CA to CPR was less than four minutes in 29 patients (65.8%), was 4–20 minutes in five patients (11.4%), and was longer than 20 minutes in three cases (6.8%). The time from arrest to resuscitation was unknown in seven instances.

VF or PVT was the first documented rhythm in 19 patients (43.2%) (10 IH and nine OOH). In the remaining 25 patients (12 IH and 13 OOH) the rhythm at the beginning of CA episode was a nonshockable rhythm (asystole in 18 cases, severe bradycardia in six cases, and pulseless electrical activity in one case), but they developed VF or PVT during the evolution of CPR.

Prior to electrical shocks, a precordial thump was performed in six patients (13.6%). None of the thumps terminated the VF or PVT. The number of shocks received by the children ranged from one to 30 (median, four shocks). Eight children (18.2%) received one shock, 11 children (25.0%) received two shocks, eight children (18.2%) received three shocks, 13 children (29.5%) received from four to six shocks, and four children (9.1%) received more than six shocks. The median number of shocks was two for IH cases and was 3.5 for OOH cases (*P *= 0.190). In total, 68.8% of OOH-arrested children needed more than three shocks, which compares with 31.3% of IH arrests (*P *= 0.116). Only 16.6% of patients who arrested in the pediatric intensive care unit needed more than three shocks, versus 47.3% of the OOH-arrested children (*P *= 0.175). Children admitted to the pediatric intensive care unit have a tendency to need fewer shocks (2.7 ± 2.1) than the rest of the patients (4.1 ± 5.4) (*P *= 0.073). The number of shocks in patients with 'initial' VF was 4.8 ± 6.6, which compares with 2.8 ± 1.3 for patients with 'secondary' VF (*P *= 0.643).

The energy delivered by the shock ranged from 1 to 12 J/kg. The mean energy dose for the first shock was 2.4 ± 1.5 J/kg, for the second shock was 3.3 ± 2.0 J/kg, and for the third shock was 4.4 ± 1.9 J/kg. The mean energy dose of the first shock in IH cases was 2.3 ± 1.2, which compares with 2.6 ± 1.7 in OOH cases (*P *= 0.770).

Forty-three out of 44 patients (97.7%) were intubated and ventilated, 40 patients (90.9%) were treated with adrenaline (range of number of doses, 1–10), and 36 patients were treated with bicarbonate (81.8%). The total CPR time was shorter than 10 minutes in five patients (11.3%), was from 10 to 30 minutes in 11 patients (25.0%), and was longer than 30 minutes in 27 patients (61.3%).

### Outcome

VF or PVT was terminated to an organized electrical rhythm with a pulse in 28 instances (63.6%). The resultant rhythm was sinus rhythm in 16 cases (36.3%), junctional rhythm in three patients (6.8%), supraventricular tachycardia in one case (2.3%), ventricular bradycardia rhythm in five patients (11.4%), and other in three patients (6.8%).

**Figure 1:**
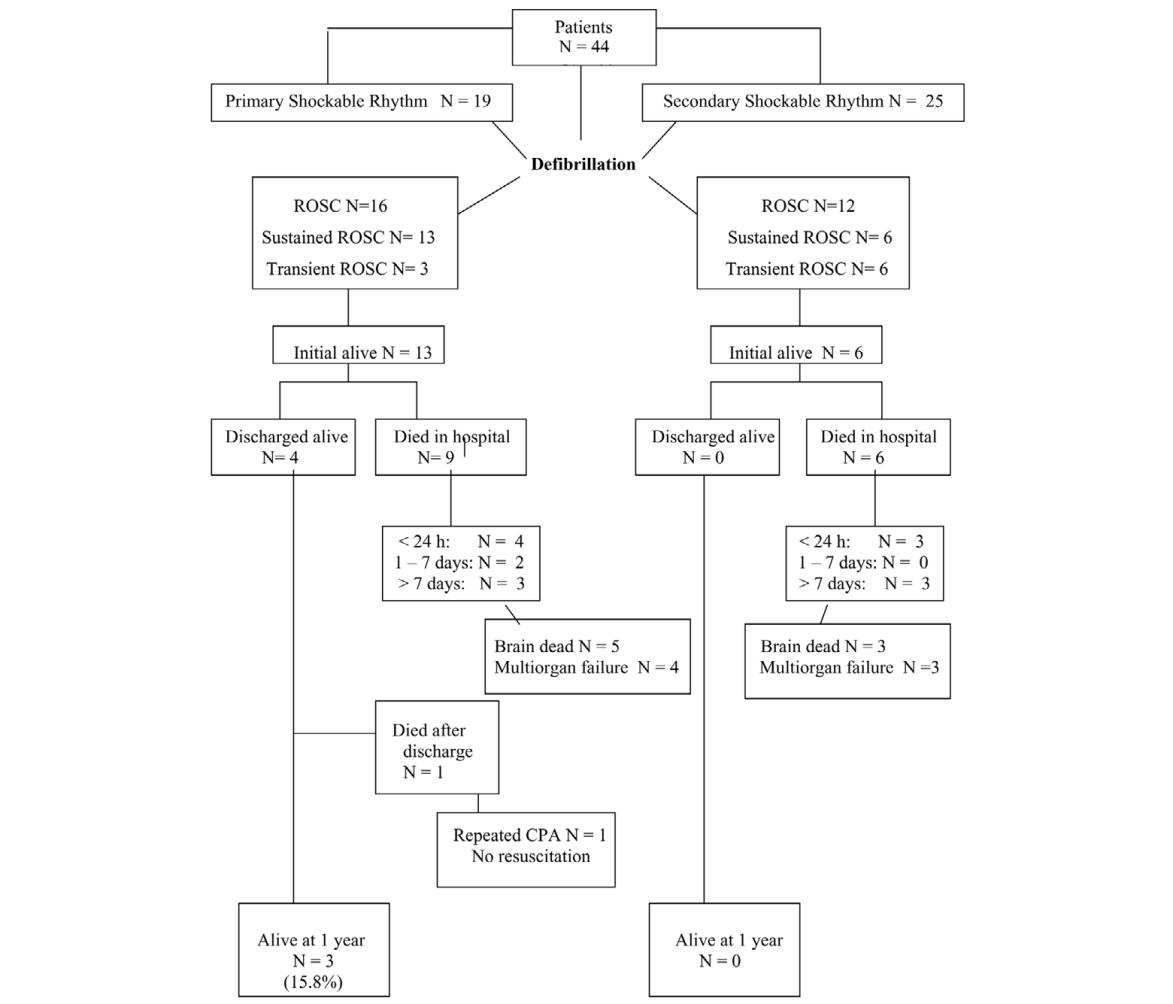
Pediatric Utstein style template for recording outcome from cardiac arrest with defibrillation. CPA= cardiopulmonary arrest; ROSC = return of spontaneous circulation.

ROSC was achieved in 28 patients (63.6%) (14 IH and 14 OOH), but the ROSC was sustained for more than 20 minutes (initial survival) only in 19 children (43.1%) (10 IH and nine OOH) (Figure 1). Of those 19 patients with ROSC >20 minutes, sixteen died later (15 during hospital stay and one after hospital discharge). The cause of death in these patients was brain death in seven cases, multiorgan failure in eight cases, and a do-not-resuscitate order in one case.

Three children (6.8%) (two IH and one OOH) survived at one year (final survival) (Figure 1). The IH-arrested and OOH-arrested children were comparable in terms of ROSC, of sustained ROSC, and of one-year survival. The neurological status and overall performance status of the three survivors, assessed by means of the pediatric cerebral performance category scale and the pediatric overall performance category scale, indicated that one patient scored 1 (normal status) in both scales at hospital discharge and at one-year follow-up, and the other two children scored 3 (moderate disability) at hospital discharge and scored 2 (mild disability) at one-year follow-up.

When groups of children were compared by the time elapsed from arrest to electric shock delivery, those undergoing a defibrillation attempt in the first four minutes had better ROSC (68.9% vs. 37.5%), better initial survival (55.1% vs. 12.5%), and better final survival (10.3% vs. 0%) than those shocked after four minutes. Statistical significance, however, was only obtained for the initial survival (*P *= 0.037) (Table 2).

Age and weight were associated with ROSC and survival. Children older than one year had better ROSC (75.0% vs. 33.0%), better initial survival (53.1% vs. 16.7%), and better final survival (9.4% vs. 0%) than infants. In this case, statistical significance was obtained only for ROSC (*P *= 0.016) and for initial survival (*P *= 0.042).

ROSC was achieved in four out of five patients with CA caused by arrhythmia, and two of these children (with congenital heart disease) were alive at one year. The other child who survived had VF secondary to hyperkalemia.

When VF or PVT was the first documented rhythm, the ROSC (84.2% vs. 48.0%), initial survival (68.4% vs. 24.0%), and final survival (15.8% vs. 0%) were higher than otherwise (Table 2).

When the electric shock dose was 2 J/kg or less, 88.6% of patients needed more than one shock; in contrast, requiring more than one shock occurred only in 42.9% of those children treated with a dose higher than 2 J/kg (*P *= 0.017). The ROSC, the sustained ROSC and the final survival, however, were similar for both groups (2 J/kg or less vs. higher than 2 J/kg dose) (Table 2).

No differences in outcome were detected when patients who received more three shocks were compared with the remaining children (Table 2). There were no statistically significant differences when the number of shocks delivered to patients with ROSC (4.1 ± 5.4) and delivered to patients without ROSC (2.8 ± 1.1) were compared (*P *= 0.856), as well as when the number of shocks to patients with final survival (2.6 ± 2.8) was compared with the number of shock to patients finally dead (3.8 ± 4.5) (*P *= 0.382). The number of shocks in patients with sustained ROSC (2.8 ± 2.2), however, was significantly lower than in patients without sustained ROSC (4.4 ± 5.6) (*P *= 0.049).

## Discussion

Early defibrillation has been recognized as an essential element in the chain of life for adults [22]. It is assumed that the same should apply for children with CA and a shockable rhythm [19]. Evidence regarding the usefulness of defibrillation in children, however, is scarce [12-14,16]. Studies that provide data about the potential effectiveness of electric energy to terminate 'shockable' rhythms that can be present in pediatric CA are therefore essential.

A recent large, multicenter, IH CA registry by the American Heart Association National Registry of CPR Investigators [12] showed that 27% of patients had documented VF or PVT during the arrest. In that study 35% of patients with initial VF or PVT survived to hospital discharge, compared with 11% of patients with subsequent VF or PVT [12]. Our prospective multicenter study, including both IH CA children and OOH CA children, also indicated that shockable rhythms can appear not only as the first documented rhythm, but can develop during CPR. In such cases, survival outcomes are very low. Explanations for poor prognosis among children with subsequent VF or PVT are not evident and could include a delay in the diagnosis of a shockable rhythm during resuscitation, adverse effects of epinephrine, or the severity of the underlying myocardial condition [12]. Whatever the causes, this is a newly recognized fact that emphasizes the need for early and continuous electrocardiogram monitoring during CPR in order to respond adequately to eventual subsequent shockable rhythms.

Although our figures compare with those reported by Berg and colleagues in a retrospective study [13], the response rate of VF/PVT to defibrillation attempts obtained in the present study is very low: nearly 82% of children did not respond to the first shock, and around 40% needed more than three shocks. In the American Heart Association registry [12], 53.1% of those patients with a known number of shocks received more than two shocks. Considering that studies in adults demonstrate that shock effectiveness is related to the time between CA and shock [22], our results are surprising because in two-thirds of cases the time from CA to shock delivery was shorter than four minutes, because 41% of children arrested when admitted to the pediatric intensive care unit, and because 61% of shockable rhythms appeared during CPR attempts. Patients with IH CA have a tendency to require fewer shocks than the other patients, however, according to the effectiveness of rapid defibrillation found in adults [22].

The pediatric defibrillation dose is mainly based on animal studies of brief-duration VF and a single pediatric study of short-duration IH VF [15,16]. Some animal studies and pediatric series, however, have suggested that doses higher than 2 J/kg are safe and could be more effective [17,18]. A study of piglets weighting 24 kg showed that biphasic energy doses of 50 J, 75 J and 84 J achieved better 24-hour survival with good neurological outcome and greater left ventricular ejection fraction than monophasic doses of 2 J/kg, 2 J/kg and 4 J/kg, although the differences were not significant in 4 kg and 14 kg piglets [17].

Pediatric studies that prospectively compare the effectiveness of low defibrillation doses versus high defibrillation doses are lacking. In this sense, our results indicate that initial or subsequent 'relatively high' doses (>2 J/kg) appear to be more effective to terminate VF or PVT than a 'low' dose. Unfortunately, it seems that termination of VF/PVT with such doses did not contribute to an increase in the immediate survival rate or final survival rate of our patients. At this point we must be cautious about making conclusions because our study was not designed to compare shock doses, because the number of patients is limited, and because other confounding factors cannot be ruled out. Experimental data on the myocardial injury provoked by electric shocks are also nonconclusive, with some studies demonstrating an absence of deleterious effects of high doses of biphasic energy [23] and other studies suggesting myocardial damage and worse neurological outcome in piglets treated with adult biphasic doses [24].

Regarding outcome, our presented results indicate that, even though almost two-thirds of patients achieved ROSC, the final survival (7%) was dismal and lower than the reported survival in adults [1-3] and in children after IH CA [3,12]. A possible explanation could be that in our sample 60% of children had VF/PVT secondary to noncardiac causes – it has been reported that VF secondary to other causes (trauma, hypoxia) probably has poorer prognosis [12]. Although perhaps anecdotal, in our series only the three patients with an arrhythmia as the direct cause of CA survived at one year.

The ROSC, the initial survival, and the final survival were slightly better in children who were defibrillated soon after arrest (in the first four minutes). This fact is not new and clearly supports the importance of early defibrillation [12,19,21].

The present study has several limitations. Although it is a prospective study following the Utstein style recommendations, it was not specifically designed to analyze the effectiveness of electric shocks or to compare different defibrillation doses. Besides, all the defibrillation devices available for use with our patients delivered monophasic waveforms. Recent studies have demonstrated that biphasic defibrillators are more efficacious than monophasic ones and are therefore recommended nowadays for adults and children [25,26].

## Conclusion

Shockable rhythms may be the first documented rhythm and also may develop subsequently during resuscitative efforts in children who suffered IH CA or OOH CA. A first shock of 2 J/kg is not effective in most of patients; therefore doses higher than 2 J/kg should be recommended from the first shock. VF/PVT termination does not assure immediate or long-term survival. Survival is better after initial VF/PVT than after subsequent VF or PVT that appear during CPR efforts. Additional prospective studies are needed in order to define the optimal dose for pediatric defibrillation.

## Key messages

• Shockable rhythms may be present in children who suffered CA.

• When VF or PVT appears during resuscitative efforts, the survival outcome is very poor.

• Monophasic shock at a dose of 2 J/kg is not effective to defibrillate children.

• Prospective registries and studies are needed to determine the optimal pediatric defibrillation procedure.

## Abbreviations

CA = cardiac arrest; CPR = cardiopulmonary resuscitation; IH = inhospital; OOH = out-of-hospital; PVT = pulseless ventricular tachycardia; ROSC = return of spontaneous circulation; VF = ventricular fibrillation.

## Competing interests

The authors declare that they have no competing interests.

## Authors' contributions

AR-N conceived and designed the study, reviewed all necessary material, and wrote the initial and successive drafts. JL-H conceived, designed and coordinated the study, analyzed the data, and critically reviewed the drafts. CG collected and analyzed the patient data. PD and AC participated in the design of the study and critically reviewed the drafts. JMB assisted in the study design and performed the statistical analysis. The study collaborators (see Appendix) were in charge of the included patients and collected the patient data. All the authors gave final approval of the version to be published.

## Appendix: study collaborators

Custodio Calvo (Hospital Materno-Infantil, Málaga), Miguel A Delgado (Pediatric Hospital, La Paz, Madrid), Corsino Rey (Asturias Central Hospital, Oviedo), María A García (Niño Jesús Hospital, Madrid), Jose A Alonso (Virgen de la Salud Hospital, Toledo), Julio Melendo (Miguel Servet Hospital, Zaragoza), Teresa Hermana (Cruces Hospital, Baracaldo), Josefina Cano (Virgen del Rocio Hospital, Sevilla), Francisco Romero (061 Emergency Service, Jaén), Servando Pantoja (Puerta del Mar Hospital, Cádiz), Carlos Lucena (061 Emergency Service, Almería), Pere Plaja (Palamós Hospital, Gerona), Ana Concheiro (San Juan de Dios Hospital, Barcelona), Alvaro Díaz (Tarrasa Hospital, Barcelona), Ricardo Martino (Príncipe de Asturias Hospital, Alcalá de Henares), María V Esteban (Princesa de España Hospital, Jaén), Nieves de Lucas (SAMUR, Madrid), Esther Ocete (Hospital Clínico, Granada), Juan I Muñoz (Reina Sofía Hospital, Córdoba), María A Rodríguez (Hospital da Barbanza, Coruña), Susana Simó (061 Emergency Service, Barcelona), Eduard Solé (Arnaú de Villanova Hospital, Lérida), Enrio Jiménez (Hospital del Mar, Barcelona), Rosario Alvarez (Jarrio Hospital, Asturias), Víctor Canduela (Laredo Hospital, Cantabria), Antonio Fernández (San Agustin Hospital, Linares), Amelia Sánchez-Galindo (Juan Canalejo Hospital, La Coruña), R Closa (Juan XXIII Hospital, Barcelona), P Villalobos (Figueras Hospital, Gerona), Orenci Urraca (Nens Hospital, Barcelona), Federico Pérez (Josep Trueta Hospital, Gerona), Antonio Torres (San Juan de Dios Hospital, Ubeda), Miguel Labay (Obispo Polanco Hospital, Teruel), M^a ^Luisa Masiques (Mollet Hospital, Barcelona), Fátima Aborto (Juan Ramón Jiménez Hospital, Huelva), Narcisa Palomino (Ciudad de Jaén Hospital, Jaén), Monserrat Miquel (San Celoni Hospital, Barcelona), Antonio Gómez Calzado (Virgen Macarena Hospital, Sevilla).

## Figures and Tables

**Table 1:** Characteristics and outcome of children who needed defibrillation

	Number of patients (%)	Return of spontaneous circulation (*n*)	*P *value	Initial survival		Final survival	
				
				*n*	*P *value	*n*	*P *value
Age			0.027		0.149		0.390
<1 month	2 (4.5%)	1		0		0	
1–12 months	9 (20.4%)	2		2		0	
1–8 years	13 (29.5%)	10		5		0	
>8 years	20 (45.4%)	15		12		3	
Gender			1		0.753		0.290
Female	16 (36.3%)	18		6		0	
Male	28 (63.6%)	10		13		3	
Site of arrest			0.827		1		0.693
Home	6 (13.6%)	5		3		0	
Public place	13 (29.5%)	7		5		1	
Emergency Department	3 (6.8%)	2		1		0	
Pediatric intensive care unit	18 (40.9%)	11		8		1	
Other hospital areas	4 (9%)	3		2		1	
Inhospital versus out-of hospital			1		1		1
Inhospital	22 (50%)	14		10		2	
Out-of-hospital	22 (50%)	14		9		1	
Diagnosis			0.256		0.484		.06
Heart disease or arrhythmia	18 (40.9%)	13		9		2	
Respiratory disease	1 (2.2%)	1		1		1	
Neurological disease	8 (18.1%)	5		3		0	
Infectious disease	3 (6.8%)	2		1		0	
Drowning	4 (9%)	3		2		0	
Sudden infant death syndrome	4 (9%)	0		0		0	
Trauma^a^	1 (2.2%)	1		0		0	
Other	2 (4.5%)	1		1		0	
Unknown	3 (6.8%)	2		2		0	

^a^Isolated head injury classified as neurological disease.

**Table 2:** Characteristics of resuscitation and outcome

	Number of patients (%)	Return of spontaneous circulation (*n*)	*P *value	Initial survival		Final survival	
				
				*n*	*P *value	*n*	*P *value
Time to initiation of cardiopulmonary resuscitation							
<4 min	29 (78.3%)	20	0.215	16	0.037	3	0.470
>4 min	8 (21.6%)	3		1		0	
First documented rhythm^a^							
Nonshockable	25 (56.8%)	12	0.025	6	0.005	0	0.073
Shockable	19 (43.1%)	16		13		3	
First shock dose							
2 J/kg	35 (83.3%)	23	0.686	15	1.0	2	0.430
>2 J/kg	7 (16.6%)	4		3		1	
Second shock dose							
2 J/kg	15 (44%)	12	0.07	9	0.01	1	0.441
>2 J/kg	19 (56%)	9		3		0	
Number of shocks							
1–3	27 (62.8%)	16	0.342	14	0.221	2	1.0
>3	16 (37.2%)	12		5		1	
Duration of cardiopulmonary resuscitation							
<10 minutes	5 (11.6%)	5	0.012	5	0.0001	1	0.067
10–30 minutes	11 (25.5%)	10		10		2	
>30 minutes	27 (62.7%)	13		4		0	

^a^Nonshockable includes asystole, bradycardia, atrioventricular block, and pulseless electrical activity; shockable includes ventricular fibrillation and pulseless ventricular tachycardia.
